# Adherence to a healthy lifestyle and all-cause and cause-specific mortality in Chinese adults: a 10-year prospective study of 0.5 million people

**DOI:** 10.1186/s12966-019-0860-z

**Published:** 2019-11-04

**Authors:** Nanbo Zhu, Canqing Yu, Yu Guo, Zheng Bian, Yuting Han, Ling Yang, Yiping Chen, Huaidong Du, Huimei Li, Fang Liu, Junshi Chen, Zhengming Chen, Jun Lv, Liming Li, on behalf of the China Kadoorie Biobank Collaborative Group, Junshi Chen, Zhengming Chen, Robert Clarke, Rory Collins, Yu Guo, Liming Li, Jun Lv, Richard Peto, Robin Walters, Daniel Avery, Ruth Boxall, Derrick Bennett, Yumei Chang, Yiping Chen, Huaidong Du, Simon Gilbert, Alex Hacker, Mike Hill, Michael Holmes, Andri Iona, Christiana Kartsonaki, Rene Kerosi, Ling Kong, Om Kurmi, Garry Lancaster, Sarah Lewington, Kuang Lin, John McDonnell, Iona Millwood, Qunhua Nie, Jayakrishnan Radhakrishnan, Paul Ryder, Sam Sansome, Dan Schmidt, Paul Sherliker, Rajani Sohoni, Becky Stevens, Iain Turnbull, Jenny Wang, Lin Wang, Neil Wright, Ling Yang, Xiaoming Yang, Zheng Bian, Xiao Han, Can Hou, Pei Pei, Chao Liu, Canqing Yu, Zengchang Pang, Ruqin Gao, Shanpeng Li, Shaojie Wang, Yongmei Liu, Ranran Du, Yajing Zang, Liang Cheng, Xiaocao Tian, Hua Zhang, Yaoming Zhai, Feng Ning, Xiaohui Sun, Feifei Li, Silu Lv, Junzheng Wang, Wei Hou, Mingyuan Zeng, Ge Jiang, Xue Zhou, Liqiu Yang, Hui He, Bo Yu, Yanjie Li, Qinai Xu, Quan Kang, Ziyan Guo, Dan Wang, Ximin Hu, Jinyan Chen, Yan Fu, Zhenwang Fu, Xiaohuan Wang, Min Weng, Zhendong Guo, Shukuan Wu, Yilei Li, Huimei Li, Zhifang Fu, Ming Wu, Yonglin Zhou, Jinyi Zhou, Ran Tao, Jie Yang, Jian Su, Fang Liu, Jun Zhang, Yihe Hu, Yan Lu, Liangcai Ma, Aiyu Tang, Shuo Zhang, Jianrong Jin, Jingchao Liu, Zhenzhu Tang, Naying Chen, Ying Huang, Mingqiang Li, Jinhuai Meng, Rong Pan, Qilian Jiang, Jian Lan, Yun Liu, Liuping Wei, Liyuan Zhou, Ningyu Chen Ping Wang, Fanwen Meng, Yulu Qin, Sisi Wang, Xianping Wu, Ningmei Zhang, Xiaofang Chen, Weiwei Zhou, Guojin Luo, Jianguo Li, Xunfu Zhong, Jiaqiu Liu, Qiang Sun, Pengfei Ge, Xiaolan Ren, Caixia Dong, Hui Zhang, Enke Mao, Xiaoping Wang, Tao Wang, Xi Zang, Ding Zhang, Gang Zhou, Shixian Feng, Liang Chang, Lei Fan, Yulian Gao, Tianyou He, Huarong Sun, Pan He, Chen Hu, Xukui Zhang, Huifang Wu, Min Yu, Ruying Hu, Hao Wang, Yijian Qian, Chunmei Wang, Kaixu Xie, Lingli Chen, Yidan Zhang, Dongxia Pan, Qijun Gu, Yuelong Huang, Biyun Chen, Li Yin, Huilin Liu, Zhongxi Fu, Qiaohua Xu, Xin Xu, Hao Zhang, Huajun Long, Xianzhi Li, Libo Zhang, Zhe Qiu

**Affiliations:** 1 Department of Epidemiology and Biostatistics, School of Public Health, Peking University Health Science Centre, 38 Xueyuan Road, Beijing, 100191 China; 2 Chinese Academy of Medical Sciences, Beijing, China; 3 Medical Research Council Population Health Research Unit at the University of Oxford, Oxford, UK; 4 Clinical Trial Service Unit & Epidemiological Studies Unit (CTSU), Nuffield Department of Population Health, University of Oxford, Oxford, UK; 5 NCDs Prevention and Control Department, Meilan Centre for Disease Control and Prevention, Haikou, Hainan China; 6 Suzhou Centre for Disease Control and Prevention, Suzhou, Jiangsu China; 7 China National Centre for Food Safety Risk Assessment, Beijing, China; 8 Key Laboratory of Molecular Cardiovascular Sciences (Peking University), Ministry of Education, Beijing, China; 9 Peking University Institute of Environmental Medicine, Beijing, China

**Keywords:** Healthy lifestyle, Mortality, Non-communicable diseases, Cohort study, Chinese

## Abstract

**Background:**

Adherence to a healthy lifestyle is associated with substantially lower risks of mortality from all causes, cardiovascular diseases, and cancer in white populations. However, little is known about the health benefits among non-white populations. Also, no previous studies have focused on respiratory disease mortality in both white and non-white populations. We assessed the relationships between a combination of healthy lifestyle factors and multiple death outcomes in Chinese adults.

**Methods:**

This study included 487,198 adults aged 30–79 years from the China Kadoorie Biobank without heart disease, stroke, and cancer at study enrolment. We defined five healthy lifestyle factors as never smoking or smoking cessation not due to illness; non-daily drinking or moderate alcohol drinking; median or higher level of physical activity; a diet rich in vegetables, fruits, legumes and fish, and limited in red meat; a body mass index of 18.5 to 27.9 kg/m^2^ and a waist circumference < 90 cm (men)/85 cm (women). Cox regression was used to produce adjusted hazard ratios (HRs) relating these healthy lifestyle factors to all-cause and cause-specific mortality.

**Results:**

During a median follow-up of 10.2 years (IQR 9.2–11.1), we documented 37,845 deaths. After multivariable adjustment, the number of healthy lifestyle factors exhibited almost inverse linear relationships with the risks of all-cause and cause-specific mortality. Compared with participants without any healthy factors, the hazard ratio of participants with five healthy factors was 0.32 [95% confidence interval (CI): 0.28, 0.37] for all-cause mortality. The corresponding HRs in specific cause of death were 0.42 (95% CI: 0.26, 0.67) for ischaemic heart disease, 0.21 (95% CI: 0.09, 0.49) for ischaemic stroke, 0.37 (95% CI: 0.22, 0.60) for haemorrhage stroke, 0.36 (95% CI: 0.29, 0.45) for cancer, 0.26 (95% CI: 0.14, 0.48) for respiratory diseases, and 0.29 (95% CI: 0.22, 0.39) for other causes. Theoretically, 38.5% (95% CI: 33.0, 43.8%) of all-cause mortality was attributable to nonadherence to a healthy lifestyle, and the proportions of preventable deaths through lifestyle modification ranged from 26.9 to 47.9% for cause-specific mortality.

**Conclusions:**

Adherence to a healthy lifestyle was associated with substantially lower risks of all-cause, cardiovascular, respiratory, and cancer mortality in Chinese adults. Promotion of a healthy lifestyle may considerably reduce the burden of non-communicable diseases in China.

## Introduction

Non-communicable diseases (NCDs), once considered “diseases of affluence”, have now encroached on low- and middle-income countries, where three quarters of worldwide NCD deaths occurred [1]. In China, more than eight out of ten deaths are caused by NCDs, with ischaemic heart disease (IHD, 15%), cerebrovascular disease (21%), cancer (23%), and chronic respiratory diseases (12%) posing the greatest threat [2]. With the development of drug therapy for NCDs, more people are living with chronic conditions. However, escalating treatment expenditure is becoming unbearable, especially for poorer countries [3]. Were we to still rely heavily on treatment, global health resources would be inadequate to tackle the growing epidemic. Hence, population-wide primary prevention targeted at reducing exposure of risk factors should be the overarching priority for the response to this global crisis.

The combined impact of lifestyle factors, including tobacco smoking, excessive alcohol intake, physical inactivity, unhealthy diet, and obesity, on mortality has been prospectively evaluated mostly in western populations [4–6]. A meta-analysis showed that a combination of at least four healthy lifestyle factors lowered the all-cause mortality risk by 66% [7]. Some studies also demonstrated considerably lower risks of cardiovascular and cancer mortality ascribed to a healthy lifestyle [8–14]. However, no prior studies have focused on respiratory disease mortality, which is thought to be closely linked to infection, occupational hazards, and ambient and household air pollution in low- and middle-income countries. It is uncertain to what extent respiratory disease mortality is attributable to unhealthy lifestyle in this population. Also, there exist racial/ethnic disparities in disease subtype composition. For example, East Asia, notably China, has higher stroke incidence and a higher proportion of haemorrhagic stroke [15]. The effectiveness of lifestyle modification strategy in NCD prevention among non-white populations remains to be investigated.

The present study used data from the China Kadoorie Biobank (CKB) study, a nationwide prospective cohort of 0.5 million adults. We aimed to examine the associations of a combination of healthy lifestyle factors with the risks of all-cause and cause-specific mortality, and estimate the proportion of deaths that could theoretically be prevented through lifestyle modification during a 10-year period. Specifically, we sought to gain an insight into the possibly different impacts on specific causes of death.

## Methods

### Study population

The CKB cohort was established during 2004–2008 in 10 geographically diverse areas of China, including five urban and five rural areas. Detailed descriptions of the CKB cohort have been previously published [16, 17]. All participants provided written informed consent, completed interviewer-administered questionnaires, and had physical measurements taken. Trained staff entered baseline information directly into a laptop-based data entry system developed with built-in functions to avoid missing items and minimise logic errors during the interview. The study received approval from the Ethical Review Committee of the Chinese Centre for Disease Control and Prevention (Beijing, China) and the Oxford Tropical Research Ethics Committee, University of Oxford (UK).

Overall, a total of 512,714 participants 30–79 years of age were eligible for inclusion. We excluded participants with self-reported clinician diagnosis of heart disease (*n* = 15,472), stroke (*n* = 8884), or cancer (*n* = 2578), and those who had missing data for body mass index (BMI, *n* = 2), leaving 487,198 participants for the current analyses. For death from respiratory diseases, we additionally excluded participants with chronic obstructive pulmonary disease (COPD, *n* = 37,055), which was ascertained based on clinical diagnosis of emphysema or bronchitis prior to the baseline survey and pulmonary function test at the time of enrolment [18], leaving 452,657 participants for this part of analysis.

### Assessment of lifestyle factors

Baseline questionnaire and physical measurement assessed the lifestyle factors of interest. Inquiry on tobacco smoking included smoking status (never, former, or current smoker); ever smokers were then asked for the frequency, type, and amount of tobacco smoked per day, and former smokers were additionally asked for years since quitting and the reason for smoking cessation. Questions about alcohol intake included typical drinking frequency (never, occasionally, monthly, weekly, or daily); drinkers with the frequency of at least once a week were further asked for the type of alcoholic beverage drunk habitually, and the volume of alcohol drunk on a typical drinking day in the past 12 months.

Information on physical activity was obtained by asking participants about their usual type and duration of activities in each of the four domains (occupational, commuting, domestic, and leisure-time) in the past 12 months. We calculated the total physical activity level by multiplying the metabolic equivalent tasks (METs) value for each activity by hours spent on that activity per day and summing the MET-hours for all activities [19] (see Additional file 1: Supplementary Material S2 and S3). Habitual dietary intakes during the past year were assessed via a validated qualitative food frequency questionnaire (see Additional file 1: Supplementary Material S4 and S5), which covered 12 major food groups in China. Each food group was provided with five frequency categories to choose from (never/rarely, monthly, 1–3 days/week, 4–6 days/week, or daily). Trained staff using calibrated instruments measured standing height, body weight, and circumferences of waist and hip. BMI was calculated as weight divided by height squared (kg/m^2^).

### Assessment of covariates

Covariate information was obtained from the baseline survey as well, including sociodemographic characteristics, personal and family medical history, and women’s reproductive information. Participants who reported at least one first-degree relative suffered from acute myocardial infarction, stroke, or cancer were considered as having a family history of corresponding disease. Prevalent hypertension was defined as measured systolic blood pressure ≥ 140 mmHg, or measured diastolic blood pressure ≥ 90 mmHg, or self-reported diagnosis of hypertension, or self-reported use of antihypertensive medication at baseline. Prevalent diabetes was defined as self-reported diabetes or screen-detected diabetes at baseline. Missing rates for menopausal status and three types of family history were < 1%, and no other exposures or covariates contained missing values. The missing values were categorised into a “missing” group when analysing.

### Definition of healthy lifestyle

We considered five lifestyle factors that have proved to be closely related to the risks of cardiovascular diseases (CVDs) and diabetes in our population [20, 21], namely, smoking, alcohol intake, physical activity, diet, and body shape. Lifestyle factors were deemed protective on the basis of previous studies, existing guidelines [22, 23], and their impacts on the present population. Healthy group regarding smoking behavior was defined in accordance with the recommendation that emphasizes the importance of not smoking and urges smoking cessation. We excluded those who had quit smoking because of illness, otherwise, it could lead to a misleadingly high risk in former smokers. The healthy group for alcohol intake comprised never-regular drinkers, weekly drinkers, and moderate daily drinkers (i.e., drinking < 30 g of pure alcohol in men and < 15 g in women per day).

For physical activity, groups were dichotomized based on the age- (< 50 years, 50–59 years, and ≥ 60 years) and sex-specific median of total physical activity level, with the higher half constituting the healthy group. For diet, based on suggestions from the Chinese Dietary Guidelines [22] and in accordance with the previous findings [24], we considered five food groups, including fresh vegetables, fresh fruits, red meat, legumes, and fish. A simple diet score was created according to the criteria as follows: eating vegetables daily, eating fruits daily, eating red meat 1–6 days per week, eating legumes ≥4 days per week, eating fish ≥1 day per week. For each food group, the participant who met the criterion received a score of 1, and otherwise received a score of 0. Therefore, the diet score ranged from 0 to 5. Participants who scored 4 to 5 were classified to be in the healthy group. For body shape, both body weight and body fat were taken into account, as a reflection of energy balance [25]. Participants having a BMI between 18.5 and 27.9 kg/m^2^ as well as a WC < 90 cm in men and < 85 cm in women constituted the healthy group [23], emphasizing avoidance of extremely high or low weight and abdominal obesity.

The number of the healthy lifestyle factors was counted, with a range from 0 (unhealthiest) to 5 (healthiest).

### Ascertainment of deaths

All ten study areas are covered by the Chinese Disease Surveillance Points system [26, 27], which provides cause-specific mortality data. Linkages with DSP death registries and local residential records, combined with annual active follow-up, were used to ascertain participants’ vital status. All deaths were coded using the 10th International Classification of Diseases by trained staff, who were blinded to baseline information. The main outcomes in the analyses were all-cause mortality, as well as mortality resulting from IHD (I20-I25), ischaemic stroke (I63), haemorrhagic stroke (I61), cancer (C00-C97), respiratory diseases (J00-J99), and all other causes.

### Statistical analysis

Person-years at risk were calculated from the date of the baseline survey to the date of death, loss to follow-up, or December 31, 2016, whichever came first. We used stratified Cox proportional hazard models, with age as the underlying time scale, to estimate the hazard ratios (HRs) and 95% confidence intervals (CIs) for the associations of individual and combined lifestyle factors with risks of all-cause and cause-specific mortality. The proportional hazards assumption was checked by comparing the HRs for the first and second half of the follow-up period.

Multivariable models were stratified jointly by study area and age at baseline in 5-year intervals, and adjusted for sex, education, marital status, hip circumference, family histories of heart attack, stroke, or cancer, and menopausal status (only in women) at baseline. When analysing individual lifestyle factors, all the lifestyle factors were included simultaneously. We included hip circumference in the model because prior studies suggested adjustment for hip circumference may allow a more precise estimation of the detrimental effects of visceral adipose tissue which was measured by waist circumference [28]. Several sensitivity analyses were conducted to examine the robustness of our results: (1) further adjusting for potential confounders including socioeconomic status (household income and occupation), or metabolic risk factors (hypertension and diabetes); (2) excluding underweight participants (BMI < 18.5 kg/m^2^); and (3) excluding deaths occurring during the first 2 years of follow-up.

To quantify the contribution of unhealthy lifestyle to the burden of disease, we calculated population attributable risk percent (PAR%), which can be interpreted as the proportional reduction in population mortality that would have occurred during follow-up if all participants had adopted a healthy lifestyle. The multivariable-adjusted PAR% and 95 CIs were estimated using a previously proposed method [29]. As PAR% is a population-specific calculation that combines both relative risks and prevalence of risk factors, we further performed stratified analyses by sex, age, education, household income, residence, family history, and baseline status of hypertension and diabetes. In sensitivity analyses, we excluded ever smokers, underweight participants, and deaths that occurred during the first 2 years of follow-up.

The calculation of PAR% was performed using SAS (version 9.4, SAS Institute Inc.), and all other statistical analyses were performed using Stata (version 15.0, StataCorp).

## Results

Of the 487,198 participants, the mean age at baseline was 51.5 years (SD 10.5) and 287,958 (59.1%) were women. Overall, the proportions of healthy group for each lifestyle factor were 70.6% for smoking, 92.9% for alcohol intake, 49.8% for physical activity, 8.5% for diet, and 71.1% for body weight and fat. Over 90% of participants had two to four healthy lifestyle factors, while only 2.1% had all five factors (Table 1). Women were more likely than men to adopt a healthy lifestyle, despite a slightly higher prevalence of obesity. With the increase in the number of healthy lifestyle factors, participants were younger, more educated, and less likely to be hypertensive or diabetic.

**Table 1:** Baseline characteristics of the study participants according to number of healthy lifestyle factors

Baseline characteristics	Number of healthy lifestyle factors^a^					
	0	1	2	3	4	5
Men (*n* = 199,240)						
No. of participants, n (%)	3173 (1.6)	26,626 (13.4)	70,911 (35.6)	73,385 (36.8)	23,575 (11.8)	1570 (0.8)
Age, year	51.9	52.2	52.5	52.3	52.2	53.2
Urban area, %	59.0	49.1	42.6	39.0	42.8	69.0
Middle school and above, %	56.5	57.5	57.1	57.3	61.0	68.6
Married, %	93.4	92.8	93.1	92.8	93.2	95.3
Prevalent hypertension, %	53.6	44.0	36.5	32.9	32.3	30.7
Prevalent diabetes, %	7.9	6.8	5.3	4.4	4.2	3.3
Family history of, %						
Heart attack	3.5	3.6	3.0	3.1	3.1	2.8
Stroke	18.3	18.8	17.7	17.5	17.7	16.6
Cancer	18.8	17.7	17.0	16.6	16.2	16.7
Dietary metrics, %						
Eating vegetables daily	93.6	94.7	94.8	94.8	95.1	99.8
Eating fruits daily	7.9	10.0	12.0	14.6	21.9	53.8
Eating red meat 1–6 days per week	50.3	57.3	62.1	65.3	71.4	89.6
Eating fish at least 1 day per week	53.6	49.9	48.2	48.0	51.7	75.8
Eating legumes at least 4 days per week	9.9	8.2	8.2	10.0	17.9	51.2
Having healthy lifestyle factors, %						
Non-smoking	–	3.2	16.0	40.5	90.9	–
Non-excessive alcohol intake	–	57.3	80.0	95.7	99.3	–
Being physically active	–	10.6	33.7	67.8	91.3	–
Healthy dietary habits	–	0.9	3.0	7.4	20.9	–
Healthy body weight and fat	–	30.7	67.6	89.2	96.9	–
Women (*n* = 287,958)						
No. of participants, n (%)	70 (< 0.1)	1725 (0.6)	45,964 (16.0)	126,861 (44.1)	104,504 (36.3)	8834 (3.1)
Age, year	59.3	60.1	54.6	51.0	49.4	49.0
Urban area, %	22.1	40.6	45.9	43.7	39.9	70.5
Middle school and above, %	30.7	30.8	39.1	43.4	44.2	55.5
Married, %	81.0	85.7	89.0	89.2	90.1	90.5
Prevalent hypertension, %	39.4	33.0	40.2	32.5	28.4	27.8
Prevalent diabetes, %	5.3	7.1	8.5	5.6	4.0	3.7
Family history of, %						
Heart attack	5.2	2.3	3.1	3.2	3.2	3.8
Stroke	21.4	16.0	17.7	17.6	17.3	18.0
Cancer	18.7	17.1	16.4	16.4	16.4	16.7
Postmenopausal, %	51.5	52.2	51.3	50.8	50.4	50.0
Dietary metrics, %						
Eating vegetables daily	94.2	94.1	94.8	94.6	94.3	99.8
Eating fruits daily	14.2	13.8	15.9	19.0	23.1	61.9
Eating red meat 1–6 days per week	52.1	62.5	62.9	65.7	71.2	89.3
Eating fish at least 1 day per week	47.7	37.2	41.2	43.4	46.2	71.4
Eating legumes at least 4 days per week	10.1	5.6	4.6	6.2	10.9	44.9
Having healthy lifestyle factors, %						
Non-smoking	–	31.1	92.8	97.8	99.9	–
Non-excessive alcohol intake	–	87.4	97.9	99.5	100.0	–
Being physically active	–	6.1	4.6	31.9	89.3	–
Healthy dietary habits	–	0.1	0.2	3.8	14.3	–
Healthy body weight and fat	–	8.2	5.2	67.1	97.1	–

All variables were adjusted for age at recruitment and survey areas, as appropriate

^a^Healthy lifestyle factors were defined as: non-smoking or having stopped for reasons other than illness; non-daily drinking or drinking < 30 g (men)/15 g (women) of pure alcohol per day; engaging in an age- (< 50 years, 50–59 years, and ≥ 60 years) and sex-specific median or higher level of physical activity; eating a diet rich in vegetables, fruits, legumes and fish, and low in red meat; having a BMI between 18.5 and 27.9 kg/m^2^ and a WC < 90 cm (men)/85 cm (women)

During a median follow-up of 10.2 years (a total of 4.8 million person-years), we documented 37,870 deaths, including 5116 from IHD, 1578 from ischaemic stroke, 4503 from haemorrhagic stroke, 12,620 from cancer, and 3655 from respiratory diseases (1833 remained after excluding participants with prevalent COPD at baseline). After adjustment for potential confounders, non-smoking, higher levels of physical activity, and higher diet score were associated with lower risk of all-cause mortality (Additional file 1: Table S1). For body shape, the lowest risk occurred in those with a BMI of 18.5–27.9 kg/m^2^ and absence of abdominal obesity; both underweight and general and/or abdominal obesity were associated with higher risk. For alcohol intake, a J-shaped association was seen, with the lowest risk of all-cause mortality for those consuming 1–15 g per day. In general, similar associations were observed for cardiovascular (Additional file 1: Table S2) and non-cardiovascular mortality (Additional file 1: Table S3), although with a few exceptions. For instance, light to moderate alcohol intake appeared to be more protective against IHD mortality, whereas heavy alcohol intake had more pronounced adverse effect on mortality from haemorrhagic stroke and cancer. Besides, the higher risk of cancer mortality was primarily seen among underweight participants, but that of stroke mortality was primarily seen among obese participants.

**Fig. 1:**
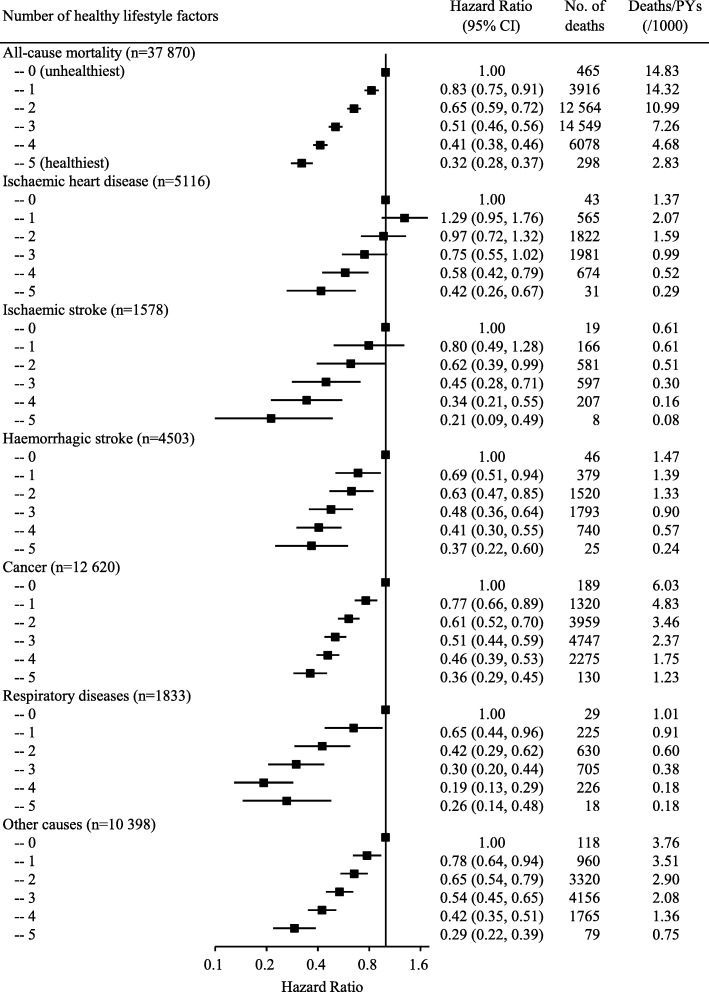
Multivariable-adjusted hazard ratios for all-cause and cause-specific mortality by number of healthy lifestyle factors. Horizontal lines represent 95% CI. ‘n’ in parentheses indicates the number of deaths. Multivariable model was adjusted for sex, education, marital status, family histories of heart attack, stroke or cancer (adjusted for in all-cause mortality and corresponding cause of death), and hip circumference. The linear trends in the risks of all outcomes with the number of healthy lifestyle factors were statistically significant (all *P* for linear trend < 0.001)

When healthy lifestyle factors were considered separately, all of them were associated with lower risk of all-cause mortality (Table 2). However, some causes of death were not significantly associated with non-smoking, non-excessive alcohol intake, or healthy dietary habits. When healthy lifestyle factors were combined, as shown in Fig. 1, the number of healthy factors exhibited almost inverse linear relationships with the risks of all-cause and cause-specific mortality (all *P* for linear trend < 0.001). In comparison with participants without any healthy factors, the adjusted HR (95% CI) of participants who had five healthy factors was 0.32 (0.28, 0.37) for all-cause mortality. The corresponding HRs in specific cause of death were 0.42 (0.26, 0.67) for IHD, 0.21 (0.09, 0.49) for ischaemic stroke, 0.37 (0.22, 0.60) for haemorrhagic stroke, 0.36 (0.29, 0.45) for cancer, 0.26 (0.14, 0.48) for respiratory diseases, and 0.29 (0.22, 0.39) for other causes. In the sensitivity analyses, further adjusting for potential confounders, and excluding underweight participants or deaths occurring in the first 2 years of follow-up did not substantially alter the risk estimates (Additional file 1: Table S4).

**Table 2:** Multivariable-adjusted hazard ratios and population attributable risk percent for all-cause and cause-specific mortality by healthy lifestyle factors

	No. of deaths in healthy group	Mortality rate per 1000 person-years in healthy group	Hazard ratio (95% CI)	Population attributable risk percent (95% CI)
All-cause mortality				
Non-smoking	21,140	6.12	0.77 (0.75, 0.79)	10.6 (9.5, 11.7)
Non-excessive alcohol intake	33,911	7.51	0.88 (0.85, 0.91)	1.3 (0.9, 1.7)
Being physically active	16,454	6.76	0.78 (0.76, 0.80)	13.9 (12.9, 15.0)
Healthy dietary habits	2233	5.43	0.89 (0.85, 0.93)	11.8 (8.0, 15.5)
Healthy body weight and fat	24,755	7.12	0.76 (0.75, 0.78)	8.2 (7.5, 8.9)
Ischaemic heart disease				
Non-smoking	3013	0.87	0.75 (0.69, 0.80)	10.4 (7.6, 13.1)
Non-excessive alcohol intake	4767	1.06	1.01 (0.90, 1.13)	NA^a^
Being physically active	1892	0.78	0.78 (0.73, 0.83)	15.3 (12.4, 18.3)
Healthy dietary habits	304	0.74	0.85 (0.75, 0.95)	15.8 (6.0, 25.2)
Healthy body weight and fat	3027	0.87	0.73 (0.68, 0.77)	11.1 (9.2, 12.9)
Ischaemic stroke				
Non-smoking	917	0.27	0.72 (0.63, 0.82)	11.6 (6.6, 16.5)
Non-excessive alcohol intake	1453	0.32	0.89 (0.73, 1.08)	0.9 (−0.7, 2.5)
Being physically active	538	0.22	0.63 (0.56, 0.71)	25.7 (20.9, 30.4)
Healthy dietary habits	91	0.22	0.87 (0.70, 1.08)	14.0 (−4.1, 31.2)
Healthy body weight and fat	988	0.28	0.81 (0.72, 0.90)	6.7 (3.1, 10.3)
Haemorrhagic stroke				
Non-smoking	2600	0.75	0.93 (0.86, 1.01)	3.5 (0.2, 6.8)
Non-excessive alcohol intake	4071	0.90	0.76 (0.68, 0.84)	2.4 (1.3, 3.5)
Being physically active	2088	0.86	0.76 (0.71, 0.81)	13.3 (10.3, 16.2)
Healthy dietary habits	149	0.36	0.78 (0.66, 0.93)	23.1 (10.3, 35.2)
Healthy body weight and fat	2975	0.86	0.82 (0.77, 0.88)	5.3 (3.2, 7.5)
Cancer				
Non-smoking	6712	1.94	0.70 (0.67, 0.74)	13.7 (11.8, 15.6)
Non-excessive alcohol intake	10,887	2.41	0.75 (0.71, 0.80)	3.4 (2.6, 4.2)
Being physically active	5984	2.46	0.90 (0.87, 0.94)	6.3 (4.5, 8.1)
Healthy dietary habits	931	2.26	0.95 (0.89, 1.02)	4.3 (−1.9, 10.5)
Healthy body weight and fat	8715	2.51	0.91 (0.87, 0.95)	2.5 (1.2, 3.8)
Respiratory diseases				
Non-smoking	982	0.30	0.70 (0.62, 0.79)	13.6 (8.8, 18.4)
Non-excessive alcohol intake	1657	0.39	0.93 (0.79, 1.10)	0.6 (−1.0, 2.3)
Being physically active	771	0.34	0.68 (0.61, 0.75)	19.8 (15.3, 24.1)
Healthy dietary habits	110	0.28	1.00 (0.81, 1.22)	2.5 (−15.8, 20.6)
Healthy body weight and fat	1074	0.33	0.56 (0.51, 0.61)	19.1 (16.8, 21.3)
Other causes				
Non-smoking	6157	1.78	0.87 (0.82, 0.91)	4.8 (2.7, 6.8)
Non-excessive alcohol intake	9392	2.08	0.95 (0.88, 1.01)	0.5 (−0.2, 1.2)
Being physically active	4467	1.83	0.76 (0.73, 0.80)	14.8 (12.8, 16.7)
Healthy dietary habits	589	1.43	0.86 (0.79, 0.93)	14.7 (7.7, 21.6)
Healthy body weight and fat	6918	1.99	0.76 (0.73, 0.79)	7.9 (6.6, 9.2)

Low-risk lifestyle factors were defined as follows: non-smoking or having stopped for reasons other than illness; non-daily drinking or drinking < 30 g (men)/15 g (women) of pure alcohol per day; engaging in an age- (< 50 years, 50–59 years, and ≥ 60 years) sex-specific median or higher level of physical activity; eating a diet rich in vegetables, fruits, legumes and fish, and low in red meat; having a BMI between 18.5 and 27.9 kg/m^2^ and a WC < 90 cm (men)/85 cm (women). Multivariable model was adjusted for sex (men or women); education (no formal school, primary school, middle school, high school, college, or university or higher); marital status (married, widowed, divorced or separated, or never married); family histories of heart attack, stroke and cancer (presence, absence, or unknown); and hip circumference (continuous). All five lifestyle factors were included simultaneously in the same model

^a^No meaningful PAR% estimate was obtained because the estimated relative risk for this factor in the model had a negative coefficient

We calculated PAR% for individual and combined lifestyle factors. With regard to all-cause mortality, the estimated PAR% (95% CI) was 10.6% (9.5, 11.7%) for tobacco smoking, 1.3% (0.9, 1.7%) for excessive alcohol intake, 13.9% (12.9, 15.0%) for lack of physical activity, 11.8% (8.0, 15.5%) for an unhealthy diet, and 8.2% (7.5, 8.9%) for extreme weight and abdominal obesity (Table 2). The combined PAR% of all-cause mortality due to smoking, lack of physical activity, and unhealthy diet was 32.2% (26.8, 37.4%), which rose up to 37.8% (32.4, 42.8%) if additionally considering extreme weight and abdominal obesity. Further inclusion of excessive alcohol intake caused little increase in PAR% (38.5%; 95% CI: 33.0, 43.8%) (Table 3). The risk attributable to all these modifiable lifestyle factors varied by specific cause of death, ranging from 26.9% (16.7, 36.6%) for cancer to 47.9% (22.7, 67.0%) for ischaemic stroke.

**Table 3:** Multivariable-adjusted population attributable risk percent for all-cause and cause-specific mortality by specific combination of healthy lifestyle factors

	Non-smoking, being physically active, healthy dietary habits	+ Healthy body weight and fat	+ Non-excessive alcohol intake
All-cause mortality	32.2 (26.8, 37.4)	37.8 (32.4, 42.8)	38.5 (33.0, 43.8)
Cause-specific mortality			
Ischaemic heart disease	36.1 (21.8, 48.9)	43.2 (29.6, 55.2)	NA^a^
Ischaemic stroke	43.7 (19.3, 62.9)	47.4 (23.0, 66.2)	47.9 (22.7, 67.0)
Haemorrhagic stroke	35.7 (15.9, 52.7)	39.1 (19.4, 55.7)	40.5 (20.5, 57.3)
Cancer	22.7 (13.2, 31.8)	24.6 (14.6, 34.2)	26.9 (16.7, 36.6)
Respiratory diseases	32.7 (7.3, 54.2)	45.4 (23.0, 63.3)	45.8 (22.5, 64.1)
Other causes	30.8 (20.2, 40.7)	36.2 (25.8, 45.8)	36.6 (25.8, 46.4)

Multivariable model was adjusted for sex, education, marital status, family histories of heart attack, stroke or cancer (adjusted for in all-cause mortality and corresponding cause of death), and hip circumference. All five lifestyle factors were included simultaneously in the same model

^a^The PAR% of ischaemic heart disease for 5 factors was not available because the estimated relative risk for excessive alcohol intake had a negative coefficient

In the subgroup analyses, broadly similar PAR% estimates were obtained from subgroups stratified according to sex, age, education, income, residence, and family history. Nevertheless, the estimated PAR% of all-cause mortality for five factors among participants with prevalent hypertension and/or diabetes at baseline was 41.1% (34.1, 47.5%), higher than those without both (32.7%; 95% CI: 23.3, 41.6%) (Additional file 1: Table S5). Repeated analysis performed among never-regular smokers exhibited lower estimates for ischaemic stroke and cancer than those obtained from the whole cohort (Additional file 1: Table S6). To further rule out the potential reverse causality, we separately excluded underweight participants and deaths that occurred in the first 2 years of follow-up, both of which did not lead to remarkable changes in PAR% estimates.

## Discussion

In this large, nationwide, prospective cohort study of Chinese adults, each of the five predefined healthy lifestyle factors was independently associated with lower risk of all-cause mortality. In combination, the total mortality risk for participants who had five healthy factors was significantly lower, compared with their counterparts in the unhealthiest group. Assuming causal relationships existed, nearly two-fifths of total deaths in this population during 10 years of follow-up, including over two-fifths of deaths from major CVDs and respiratory diseases, one quarter of deaths from cancer, and one-third of deaths from other causes, could have been prevented through lifestyle modification. The proportion of theoretically preventable deaths was even higher among participants with hypertension and/or diabetes.

Consistent with previous studies [4–6, 8–14], our findings indicated a significant inverse relationship between the number of healthy lifestyle factors and all-cause mortality. However, mortality burden attributable to an unhealthy lifestyle varied across studies. Pooled analysis of the Nurses’ Health Study and the Health Professionals Follow-up Study showed that the PAR% of nonadherence to never smoking, moderate alcohol intake, moderate to vigorous physical activity, a healthy dietary pattern, and an optimal body weight was 60.7% (53.6, 66.7%) for all-cause mortality [14]. Similar considerable proportional reduction in all-cause mortality was estimated in British and European cohorts [8, 11]. However, findings from the Asian population exhibited lower estimates for all-cause mortality, about 30% in women and 45% in men [30–33], which approximated to our results. Proposed explanations for this Asian-white difference included differences in lifestyle and genetic factors. In addition, infection, occupational and environmental exposure are still major causes of disease burden in low- and middle-income countries.

In the present study, the mortality burden attributable to an unhealthy lifestyle was higher for CVD than for cancer, which is in line with most previous findings [11, 12, 14, 32]. It was estimated that the PAR% of nonadherence to 5 healthy lifestyle factors was 71.7% (58.1, 81.0%) for CVD mortality in the US population, in contrast to 51.7% (37.1, 62.9%) for cancer mortality [14]. These lifestyle factors common to CVD and cancer, however, yielded different magnitude of effects on each type of disease. Environmental carcinogens and some infectious agents, as key underlying causes for cancer, may also result in the lower PAR% estimate for cancer mortality.

Few studies have addressed the associations between a combination of lifestyle factors and stroke mortality. The Singapore Chinese Health Study observed 75% lower risk of cerebrovascular disease mortality among individuals with ≥5 protective lifestyle factors [34]. Findings from the Japan Collaborative Cohort Study showed the PAR% was 45.0 and 43.4% in men and women, respectively [35]. Due to limited sample sizes, analyses were not performed separately for ischaemic and haemorrhagic stroke in these two studies. Our study which further identified stroke subtypes for the first time showed that despite the differences in pathogenesis, the proportion of deaths attributable to an unhealthy lifestyle were generally similar between ischaemic stroke and haemorrhagic stroke.

Earlier studies showed a healthy lifestyle also protected against non-CVD non-cancer mortality [8, 10–12]. To our knowledge, this is the first study to investigate the combined effects of lifestyle factors on respiratory disease mortality, one of the major contributors to the global burden of disease. Nearly half of the deaths from respiratory diseases might have been prevented through adopting a healthy lifestyle in the present Chinese population. Also, in the present study, infectious diseases and external causes (e.g., injury) accounted for one-third of the deaths from other causes. The strong gradient for the lower risk of mortality from other causes suggested the favourable effects of a healthy lifestyle may be not limited to NCDs.

Of note, the healthy group for alcohol intake comprised only moderate drinkers in some previous studies [5, 8, 10, 12, 14, 34], while the present analyses included both non-daily drinkers and moderate drinkers. The application of different definitions would generate distinctive estimates of alcohol-attributable burden. Yet, our data showed that the cardioprotective effect of light to moderate alcohol intake was counterbalanced by higher risk of cancer mortality. Recently, the Global Burden of Disease Study 2016 reexamined the complex association of alcohol intake with health and concluded that consuming zero standard drinks daily minimized the overall health risk [36], pointing to a need to revisit current alcohol control policies. Given the relatively lower prevalence of regular drinking in the Chinese population, especially among women, encouraging moderate intake of alcohol for abstainers would have an extensive and uncertain influence. Careful deliberations are required when making recommendations about alcohol intake.

The chief strengths of our study include its prospective design, large sample size and a large number of accumulated death cases, which enabled us to comprehensively assess the relationships between a combination of lifestyle factors and multiple death outcomes. The inclusion of a geographically dispersed and socioeconomically diverse population permitted us to examine the associations across several important subgroups such as residence, sex, age and education, which makes our results more informative.

This study was also subject to several limitations. Causal inference with observational data is susceptible to several possible sources of bias. Some measurement errors were inevitable since the lifestyle behaviours were self-reported. No separate validation of the questions on physical activity by comparing directly with a reference method has been undertaken; however, these questions were adapted from validated questionnaires used in several other studies, with some additional modifications after a pilot study. Such misclassification should be nondifferential in prospective studies and tends to attenuate the associations. The present analyses only used information on lifestyle factors collected at baseline, and could not necessarily account for the impact of long-term lifestyle patterns. We excluded participants with prior major chronic diseases, and participants who might be in poor health conditions manifested by underweight and dying soon after the start of follow-up. Though carefully dealt with, reverse causality may be still present. Besides, residual confounding due to unmeasured or unknown factors cannot be completely ruled out in observational research. In developing countries including China, physical activity is derived mainly from occupation and housework [37], which together made up nearly 90% of daily physical activity in the CKB population [38]. Therefore, we defined the healthy group using the total daily physical activity level, instead of the goal levels recommended by current guidelines which were based on leisure-time physical activity. Detailed dietary information was unavailable, and consequently the association between dietary factors and mortality may be underestimated.

## Conclusions

This large prospective cohort study of Chinese adults confirmed that a substantial reduction in the burden of cardiovascular, respiratory diseases and cancer could be achieved by adherence to a healthy lifestyle pattern. If all participants had followed this lifestyle pattern, approximately two-fifths of total deaths during 10 years of follow-up could have been prevented. In light of rapid population aging and constrained medical resources in China and other developing countries, cost-effective population-wide lifestyle interventions that are affordable for most countries could be a better way to respond to the challenges posed by NCDs.

## Supplementary information

**Additional file 1:** Supplementary material and tables.

## Data Availability

The dataset supporting the conclusions of this article is available from the study website (http://www.ckbiobank.org), along with the access policy and procedures.

## Abbreviations

BMI: Body mass index
CI: Confidence interval
CKB: China Kadoorie Biobank
COPD: Chronic obstructive pulmonary disease
CVD: Cardiovascular diseases
HR: Hazard ratio
IHD: Ischaemic heart disease
MET: Metabolic equivalent task
NCD: Non-communicable disease
PAR%: Population attributable risk percent
WC: Waist circumference
