# Supplementary material for: Adherence to a healthy lifestyle and all-cause and cause-specific mortality in Chinese adults: a 10-year prospective study of 0.5 million people
Source: Int J Behav Nutr Phys Act. 2019 Nov 4;16:98. doi: 10.1186/s12966-019-0860-z (PMC6827204; doi:10.1186/s12966-019-0860-z)
Supplement: Supplementary file 1 — Additional file 1: Supplementary material and tables. [file 12966_2019_860_MOESM1_ESM.docx]

**Supplementary Appendix**

[**Supplementary Material S1. Members of the China Kadoorie Biobank collaborative group** 2](#_Toc23242021)

[**Supplementary Material S2. Questionnaire on physical activity used in the CKB study at baseline** 4](#_Toc23242022)

[**Supplementary Material S3. Physical activity types, MET values, codes and intensity categories** 6](#_Toc23242023)

[**Supplementary Material S4. Food frequency questionnaire (FFQ) used in the CKB study at baseline** 8](#_Toc23242024)

[**Supplementary Material S5. Validation of food frequency questionnaire** 8](#_Toc23242025)

[**Table S1. Multivariable-adjusted hazard ratios for all-cause mortality by lifestyle factors** 9](#_Toc23242026)

[**Table S2. Multivariable-adjusted hazard ratios for cardiovascular mortality by lifestyle factors** 11](#_Toc23242027)

[**Table S3. Multivariable-adjusted hazard ratios for non-cardiovascular mortality by lifestyle factors** 13](#_Toc23242028)

[**Table S4. Multivariable-adjusted hazard ratios for all-cause and cause-specific mortality by number of healthy lifestyle factors after additional adjustments or further exclusions** 15](#_Toc23242029)

[**Table S5. Subgroup analysis of population attributable risk percent for all-cause mortality by specific combination of healthy lifestyle factors** 18](#_Toc23242030)

[**Table S6. Multivariable-adjusted population attributable risk percent for all-cause and cause-specific mortality by specific combination of healthy lifestyle factors among some subpopulations** 19](#_Toc23242031)

**Supplementary Material S1. Members of the China Kadoorie Biobank collaborative group**

**International Steering Committee:** Junshi Chen, Zhengming Chen (PI), Robert Clarke, Rory Collins, Yu Guo, Liming Li (PI), Jun Lv, Richard Peto, Robin Walters. **International Co-ordinating Centre, Oxford:** Daniel Avery, Ruth Boxall, Derrick Bennett, Yumei Chang, Yiping Chen, Zhengming Chen, Robert Clarke, Huaidong Du, Simon Gilbert, Alex Hacker, Mike Hill, Michael Holmes, Andri Iona, Christiana Kartsonaki, Rene Kerosi, Ling Kong, Om Kurmi, Garry Lancaster, Sarah Lewington, Kuang Lin, John McDonnell, Iona Millwood, Qunhua Nie, Jayakrishnan Radhakrishnan, Paul Ryder, Sam Sansome, Dan Schmidt, Paul Sherliker, Rajani Sohoni, Becky Stevens, Iain Turnbull, Robin Walters, Jenny Wang, Lin Wang, Neil Wright, Ling Yang, Xiaoming Yang. **National Co-ordinating Centre, Beijing:** Zheng Bian, Yu Guo, Xiao Han, Can Hou, Jun Lv, Pei Pei, Chao Liu, Canqing Yu. **10 Regional Co-ordinating Centres: Qingdao CDC:** Zengchang Pang, Ruqin Gao, Shanpeng Li, Shaojie Wang, Yongmei Liu, Ranran Du, Yajing Zang, Liang Cheng, Xiaocao Tian, Hua Zhang, Yaoming Zhai, Feng Ning, Xiaohui Sun, Feifei Li. **Licang CDC:** Silu Lv, Junzheng Wang, Wei Hou. **Heilongjiang Provincial CDC:** Mingyuan Zeng, Ge Jiang, Xue Zhou. **Nangang CDC:** Liqiu Yang, Hui He, Bo Yu, Yanjie Li, Qinai Xu,Quan Kang, Ziyan Guo. **Hainan Provincial CDC:** Dan Wang, Ximin Hu, Jinyan Chen, Yan Fu, Zhenwang Fu, Xiaohuan Wang. **Meilan CDC:** Min Weng, Zhendong Guo, Shukuan Wu,Yilei Li, Huimei Li, Zhifang Fu. **Jiangsu Provincial CDC:** Ming Wu, Yonglin Zhou, Jinyi Zhou, Ran Tao, Jie Yang, Jian Su. **Suzhou CDC:** Fang liu, Jun Zhang, Yihe Hu, Yan Lu, Liangcai Ma, Aiyu Tang, Shuo Zhang, Jianrong Jin, Jingchao Liu. **Guangxi Provincial CDC:** Zhenzhu Tang, Naying Chen, Ying Huang. **Liuzhou CDC:** Mingqiang Li, Jinhuai Meng, Rong Pan, Qilian Jiang, Jian Lan,Yun Liu, Liuping Wei, Liyuan Zhou, Ningyu Chen Ping Wang, Fanwen Meng, Yulu Qin,, Sisi Wang. **Sichuan Provincial CDC:** Xianping Wu, Ningmei Zhang, Xiaofang Chen,Weiwei Zhou. **Pengzhou CDC:** Guojin Luo, Jianguo Li, Xiaofang Chen, Xunfu Zhong, Jiaqiu Liu, Qiang Sun. **Gansu Provincial CDC:** Pengfei Ge, Xiaolan Ren, Caixia Dong. **Maiji CDC:** Hui Zhang, Enke Mao, Xiaoping Wang, Tao Wang, Xi zhang. **Henan Provincial CDC:** Ding Zhang, Gang Zhou, Shixian Feng, Liang Chang, Lei Fan. **Huixian CDC:** Yulian Gao, Tianyou He, Huarong Sun, Pan He, Chen Hu, Xukui Zhang, Huifang Wu, Pan He. **Zhejiang Provincial CDC:** Min Yu, Ruying Hu, Hao Wang. Tongxiang CDC: Yijian Qian, Chunmei Wang, Kaixu Xie, Lingli Chen, Yidan Zhang, Dongxia Pan, Qijun Gu. **Hunan Provincial CDC:** Yuelong Huang, Biyun Chen, Li Yin, Huilin Liu, Zhongxi Fu, Qiaohua Xu. **Liuyang CDC:** Xin Xu, Hao Zhang, Huajun Long, Xianzhi Li, Libo Zhang, Zhe Qiu.

**Supplementary Material S2. Questionnaire on physical activity used in the CKB study at baseline**

- **Section A: For non-farmers**

**1. In the past 12 months, how active were you at work?**

🞎 Mainly sedentary (e.g. office worker)

🞎 Standing occupation (e.g. guard, shop assistant)

🞎 Manual work (e.g. plumber, carpenter)

🞎 Heavy manual work (e.g. miner, construction worker)

🞎 Retired, housewife/husband, unemployed, or disabled **🡪 *go to Question 12***

**2. In a typical week, about how many hours did you usually work?**  hours

**3. In the past 12 months, how did you usually get to work?**

🞎 Mainly walk 🞎 By bicycle

🞎 By motorbike 🞎 By bus/car/ferry/train

🞎 Mainly stayed at home, or work near home **🡪 *go to Question 12***

**4. How much time did you spend each day on the journey to and from work?**  mins

- **Section B: For farmers**

**5. In the past 12 months, did your farming work change seasonally?**

🞎 No **🡪 *go to Question 7***

🞎 Yes

**6. During the farming season in the last 12 months:**

- How many months did it usually last? months
- What types of work did it usually involve?

🞎 Manual 🞎 Semi-mechanized 🞎 Fully mechanized

- How many hours did you usually work each day? hours
- Of which, how many hours did you sweat or have a much faster heartbeat?

hours

**7. In a typical week, how many hours did you usually work in the field?**  hours

**8. Apart from the agriculture work, did you have any other job?**

🞎 No **🡪 *go to Question 11***

🞎 Yes

**9. How active were you at work with other job?**

🞎 Mainly sedentary 🞎 Mainly general manual work

🞎 Mainly standing 🞎 Mainly heavy manual work

**10. In a typical week, about how many hours did you work at other job?**  hours

**11. In a typical day how much time did you usually spend on the journey to and from work on foot or by bicycle?**  mins

- **Section C: For both farmers and non-farmers**

**12. During the past 12 months, how often did you exercise in your leisure time?**

🞎 Never or almost never

🡪 ***go to Question 15***

🞎 1-3 times/month

🞎 1-2 times/week

🞎 3-5 times/week

🞎 Daily or almost every day

**13. What is your main type of exercise? *(tick one box only)***

🞎 Taichi/Qigong 🞎 Walking

🞎 Jogging/aerobic exercise 🞎 Swimming

🞎 Ball games (basketball, table tennis, etc.) 🞎 Other (eg. mountain climbing)

**14. About how many hours per week did you spent on these exercises?** ____hours

**15. In a typical week during the past 12 months, how often did you sweat or have a much faster heartbeat because of physical activities/exercise?**

🞎 Never or almost never

**🡪 *go to Question 17***

🞎 <1 time/week

🞎 1-2 times/week

🞎 3-5 times/week

🞎 Daily or almost every day

**16. About how many hours per week did you do such vigorous activities?** ____hours

**17. About how many hours per week did you do housework?** ____ hours

**Supplementary Material S3. Physical activity types, MET values, codes and intensity categories**

| Activity type | Intensity | MET | Codes^*^ |
| --- | --- | --- | --- |
| Heavy manual work | Vigorous | 6.5 | 11477 |
| Manual work | Moderate | 4.5 | 11476 |
| Standing work | Moderate | 3.8 | Mean of 11610 and 11630 |
| Sedentary work | Low | 1.8 | Mean of 11580, 11585, and 11590 |
| Manual work in the farming season | Vigorous | 6.3 | Mean of 11145 and 11146 |
| Semi-mechanized work in the farming season | Moderate | 3.4 | Mean of 11146 and 11147 |
| Fully mechanized work in the farming season | Low | 2.4 | Mean of 11147 and 11170 |
| Work outside the farming season | Low | 2.0 | 11147 |
| Walking | Moderate | 4.0 | 17270 |
| Bicycle | Vigorous | 6.8 | 1011 |
| Motorbike | Moderate | 3.5 | 16030 |
| Private or public transportation (such as bus, car, underground, and ferry) | Low | 1.7 | Mean of 16010, 16015, and 16016 |
| Household activity | Low | 2.8 | Mean of 05030^†^, 05040^†^, 05035, 05055, 05070, 05090^†^, 05092^†^, 05184, 05197, and 05200 |
| Tai-Chi/qigong/leisure walking | Moderate | 3.3 | Mean of 15670 and 17160 |
| Jogging/aerobic exercise | Vigorous | 7.4 | Mean of 03015, 12020, and 12150 |
| Ball games | Moderate | 5.5 | Mean of 15020^†^, 15030^†^, 15055, 15080, 15090, 15255, 15605^†^, 15610^†^, 15652, 15660, 15675, 15710^†^, and 15711^†^ |
| Brisk walking/gymnastics/folk dancing | Moderate | 4.2 | Mean of 03025, 15300, and 17200 |
| Swimming | Vigorous | 7.2 | Mean of 18230, 18240, and 18310 |
| Other exercise, e.g. mountain walking, home exercise and rope jumping | Moderate | 5.9 | Mean of 02010, 02064, 04001, 04100, 15110^†^, 15120^†^, 15200, 15240, 15310, 15425^†^, 15430^†^, 15537, 15550^‡^, 15551^‡^, 15552^‡^, 15580, 15590, 15730, 15732^‡^, 15733^‡^, 15734^‡^, and 19030 |

MET, metabolic equivalent of task.

^*^Based on the 2011 Compendium of Physical Activities: a second update of codes and MET values. Ainsworth BE, et al. Medicine and Science in Sports and Exercise, 2011;43(8):1575-1581.

^†^Assigned 1/2 weight in calculating the mean MET value because the connecting two items represent one type of activity.

^‡^Assigned 1/3 weight in calculating the mean MET value because the connecting three items represent one type of activity.

**Supplementary Material S4. Food frequency questionnaire (FFQ) used in the CKB study at baseline**

- **During the past 12 months, about how often did you eat the following foods?**

|  | Daily | 4-6 days/week | 1-3 days/week | Monthly | Never/rarely |
| --- | --- | --- | --- | --- | --- |
| Rice | □ | □ | □ | □ | □ |
| Wheat | □ | □ | □ | □ | □ |
| Other staple food (corn, millet, etc.) | □ | □ | □ | □ | □ |
| Meat | □ | □ | □ | □ | □ |
| Poultry | □ | □ | □ | □ | □ |
| Fish/sea food | □ | □ | □ | □ | □ |
| Fresh eggs | □ | □ | □ | □ | □ |
| Fresh vegetables | □ | □ | □ | □ | □ |
| Soybean products | □ | □ | □ | □ | □ |
| Preserved vegetables | □ | □ | □ | □ | □ |
| Fresh fruit | □ | □ | □ | □ | □ |
| Dairy products (milk, yogurt) | □ | □ | □ | □ | □ |

**Supplementary Material S5. Validation of food frequency questionnaire**

To evaluate the reproducibility and relative validity of baseline FFQ, during 2015-2016, 432 participants were selected from four survey sites of the CKB study. The validity analysis of baseline FFQ used 24-hour dietary recalls as a reference, and the reproducibility was verified by twice administrations of FFQ. In both evaluations, weighted kappa coefficients exceeded 0.60 for all food groups except fresh vegetable. The low kappa of fresh vegetable might be due to its high proportion of agreements on daily intake, instead, the percentage of correct classification was over 89% and the percentage of extreme classification was below 1% for both validity and reproducibility. Therefore, validation study indicated that the FFQ estimated the frequency of dietary intakes with reasonably good validity and reproducibility.

**Table S1. Multivariable-adjusted hazard ratios for all-cause mortality by lifestyle factors**

|  | **No. of deaths** | **Mortality rate per 1000 person-years** | **Hazard ratio (95% CI)** | **P_trend_^*^** |
| --- | --- | --- | --- | --- |
| **Smoking** |  |  |  |  |
| Never | 19 433 | 5.86 | 1.00 | — |
| Former | 1707 | 12.37 | 1.12 (1.06, 1.18) | — |
| Current (cigarettes or equivalents/day) |  |  |  |  |
| <15 | 6982 | 13.71 | 1.28 (1.24, 1.32) | <0.001 |
| 15–24 | 6787 | 10.59 | 1.35 (1.31, 1.40) |  |
| ≥25 | 2961 | 11.79 | 1.43 (1.36, 1.49) |  |
| **Alcohol intake** |  |  |  |  |
| Non-daily | 32 741 | 7.41 | 1.00 | — |
| Current daily (g of pure alcohol/day) |  |  |  |  |
| <15 | 291 | 12.53 | 0.86 (0.77, 0.97) | <0.001 |
| 15–29 | 963 | 11.44 | 0.98 (0.92, 1.05) |  |
| 30–59 | 1607 | 11.19 | 1.04 (0.99, 1.09) |  |
| ≥60 | 2268 | 12.00 | 1.22 (1.16, 1.27) |  |
| **Physical activity** |  |  |  |  |
| Quintile 1 | 9851 | 10.22 | 1.00 | <0.001 |
| Quintile 2 | 7855 | 8.10 | 0.82 (0.80, 0.85) |  |
| Quintile 3 | 7128 | 7.38 | 0.74 (0.72, 0.77) |  |
| Quintile 4 | 6904 | 7.09 | 0.70 (0.67, 0.72) |  |
| Quintile 5 | 6132 | 6.24 | 0.63 (0.61, 0.66) |  |
| **Diet score** |  |  |  |  |
| 0–1 | 6292 | 10.24 | 1.00 | <0.001 |
| 2 | 19 519 | 8.40 | 0.92 (0.89, 0.95) |  |
| 3 | 9826 | 6.52 | 0.86 (0.83, 0.90) |  |
| 4 | 2018 | 5.44 | 0.80 (0.76, 0.85) |  |
| 5 | 215 | 5.34 | 0.77 (0.68, 0.89) |  |
| **Body weight and fat** |  |  |  |  |
| BMI <18.5 | 3900 | 19.28 | 1.37 (1.32, 1.43) | — |
| BMI 18.5–27.9, WC <90(M)/85(F) | 24 755 | 7.12 | 1.00 | — |
| BMI 18.5–27.9, WC ≥90(M)/85(F) | 5771 | 8.52 | 1.20 (1.16, 1.24) | — |
| BMI ≥28.0, WC <90(M)/85(F) | 274 | 4.98 | 1.21 (1.08, 1.37) | — |
| BMI ≥28.0, WC ≥90(M)/85(F) | 3170 | 7.16 | 1.40 (1.34, 1.47) | — |

Body mass index (BMI) unit is kg/m², waist circumference (WC) unit is cm.

MET, metabolic equivalent task.

Multivariable model was adjusted for sex (men or women); education (no formal school, primary school, middle school, high school, college, or university or higher); marital status (married, widowed, divorced or separated, or never married); family histories of heart attack, stroke and cancer (presence, absence, or unknown); and hip circumference (continuous). All five lifestyle factors were included simultaneously in the same model.

^*^The linear trend test for smoking was performed only in current smokers, and that for alcohol intake was performed only in current daily drinkers.

**Table S2. Multivariable-adjusted hazard ratios for cardiovascular mortality by lifestyle factors**

|  | **Ischaemic heart disease** | | |  | **Ischaemic stroke** | | |  | **Haemorrhagic stroke** | | |
| --- | --- | --- | --- | --- | --- | --- | --- | --- | --- | --- | --- |
|  | **No. of deaths** | **Deaths/PYs (/1,000)** | **Hazard ratio (95% CI)** |  | **No. of deaths** | **Deaths/PYs (/1,000)** | **Hazard ratio (95% CI)** |  | **No. of deaths** | **Deaths/PYs (/1,000)** | **Hazard ratio (95% CI)** |
| **Smoking** |  |  |  |  |  |  |  |  |  |  |  |
| Never | 2,770 | 0.84 | 1.00 |  | 843 | 0.25 | 1.00 |  | 2,436 | 0.73 | 1.00 |
| Former | 243 | 1.76 | 1.11 (0.97, 1.29) |  | 74 | 0.54 | 1.04 (0.80, 1.34) |  | 164 | 1.19 | 1.00 (0.84, 1.18) |
| Current (cigarettes or equivalents/day) |  |  |  |  |  |  |  |  |  |  |  |
| <15 | 966 | 1.90 | 1.28 (1.17, 1.40) |  | 320 | 0.63 | 1.39 (1.19, 1.63) |  | 878 | 1.72 | 1.11 (1.01, 1.22) |
| 15-24 | 841 | 1.31 | 1.48 (1.34, 1.63) |  | 247 | 0.39 | 1.39 (1.16, 1.66) |  | 704 | 1.10 | 1.02 (0.92, 1.14) |
| ≥25 | 296 | 1.18 | 1.48 (1.28, 1.70) |  | 94 | 0.37 | 1.53 (1.20, 1.96) |  | 321 | 1.28 | 1.09 (0.95, 1.26) |
| **Alcohol intake** |  |  |  |  |  |  |  |  |  |  |  |
| Non-daily | 4,635 | 1.05 | 1.00 |  | 1,407 | 0.32 | 1.00 |  | 3,967 | 0.90 | 1.00 |
| Current daily (g of pure alcohol/day) |  |  |  |  |  |  |  |  |  |  |  |
| <15 | 40 | 1.72 | 0.80 (0.59, 1.10) |  | 9 | 0.39 | 0.53 (0.27, 1.01) |  | 22 | 0.95 | 0.87 (0.57, 1.32) |
| 15-29 | 104 | 1.24 | 0.78 (0.64, 0.95) |  | 40 | 0.48 | 0.99 (0.72, 1.37) |  | 94 | 1.12 | 1.09 (0.88, 1.34) |
| 30-59 | 161 | 1.12 | 0.88 (0.75, 1.04) |  | 70 | 0.49 | 1.24 (0.97, 1.59) |  | 176 | 1.23 | 1.31 (1.12, 1.53) |
| ≥60 | 176 | 0.93 | 1.03 (0.88, 1.21) |  | 52 | 0.28 | 0.98 (0.73, 1.31) |  | 244 | 1.29 | 1.37 (1.19, 1.57) |
| **Physical activity** |  |  |  |  |  |  |  |  |  |  |  |
| Quintile 1 | 1,537 | 1.59 | 1.00 |  | 511 | 0.53 | 1.00 |  | 1,071 | 1.11 | 1.00 |
| Quintile 2 | 1,149 | 1.19 | 0.79 (0.73, 0.86) |  | 348 | 0.36 | 0.78 (0.68, 0.90) |  | 897 | 0.93 | 0.78 (0.72, 0.86) |
| Quintile 3 | 994 | 1.03 | 0.73 (0.67, 0.79) |  | 301 | 0.31 | 0.72 (0.62, 0.83) |  | 807 | 0.84 | 0.69 (0.63, 0.76) |
| Quintile 4 | 856 | 0.88 | 0.71 (0.65, 0.77) |  | 238 | 0.24 | 0.59 (0.50, 0.70) |  | 861 | 0.88 | 0.66 (0.60, 0.72) |
| Quintile 5 | 580 | 0.59 | 0.59 (0.53, 0.66) |  | 180 | 0.18 | 0.48 (0.39, 0.57) |  | 867 | 0.88 | 0.64 (0.58, 0.70) |
| **Diet score** |  |  |  |  |  |  |  |  |  |  |  |
| 0-1 | 954 | 1.55 | 1.00 |  | 317 | 0.52 | 1.00 |  | 1,008 | 1.64 | 1.00 |
| 2 | 2,616 | 1.13 | 0.92 (0.85, 0.99) |  | 788 | 0.34 | 0.80 (0.70, 0.92) |  | 2,416 | 1.04 | 0.90 (0.83, 0.98) |
| 3 | 1,242 | 0.82 | 0.85 (0.77, 0.94) |  | 382 | 0.25 | 0.72 (0.61, 0.86) |  | 930 | 0.62 | 0.81 (0.73, 0.90) |
| 4 | 273 | 0.74 | 0.76 (0.66, 0.88) |  | 80 | 0.22 | 0.67 (0.52, 0.87) |  | 139 | 0.37 | 0.70 (0.58, 0.84) |
| 5 | 31 | 0.77 | 0.74 (0.51, 1.06) |  | 11 | 0.27 | 0.77 (0.42, 1.42) |  | 10 | 0.25 | 0.54 (0.29, 1.02) |
| **Body weight and fat** |  |  |  |  |  |  |  |  |  |  |  |
| BMI <18.5 | 472 | 2.33 | 1.26 (1.14, 1.41) |  | 138 | 0.68 | 1.04 (0.86, 1.26) |  | 391 | 1.93 | 0.95 (0.85, 1.07) |
| BMI 18.5-27.9, WC <90(M)/85(F) | 3,027 | 0.87 | 1.00 |  | 988 | 0.28 | 1.00 |  | 2,975 | 0.86 | 1.00 |
| BMI 18.5-27.9, WC ≥90(M)/85(F) | 1,004 | 1.48 | 1.38 (1.27, 1.50) |  | 274 | 0.40 | 1.24 (1.06, 1.45) |  | 718 | 1.06 | 1.31 (1.19, 1.44) |
| BMI ≥28.0, WC <90(M)/85(F) | 35 | 0.64 | 1.28 (0.92, 1.80) |  | 15 | 0.27 | 1.81 (1.08, 3.03) |  | 30 | 0.54 | 1.46 (1.02, 2.11) |
| BMI ≥28.0, WC ≥90(M)/85(F) | 578 | 1.31 | 1.65 (1.47, 1.86) |  | 163 | 0.37 | 1.71 (1.38, 2.13) |  | 389 | 0.88 | 1.77 (1.55, 2.03) |

Abbreviations: PY, person-year; BMI, body mass index; WC, waist circumference.

Multivariable model was adjusted for sex, education, marital status, family histories of heart attack or stroke (adjusted for in the corresponding cause of death), and hip circumference at baseline. All five lifestyle factors were included simultaneously in the same model.

**Table S3. Multivariable-adjusted hazard ratios for non-cardiovascular mortality by lifestyle factors**

|  | **Cancer** | | |  | **Respiratory diseases** | | |  | **Other causes** | | |
| --- | --- | --- | --- | --- | --- | --- | --- | --- | --- | --- | --- |
|  | **No. of deaths** | **Deaths/PYs (/1,000)** | **Hazard ratio (95% CI)** |  | **No. of deaths** | **Deaths/PYs (/1,000)** | **Hazard ratio (95% CI)** |  | **No. of deaths** | **Deaths/PYs (/1,000)** | **Hazard ratio (95% CI)** |
| **Smoking** |  |  |  |  |  |  |  |  |  |  |  |
| Never | 6,135 | 1.85 | 1.00 |  | 887 | 0.28 | 1.00 |  | 5,695 | 1.72 | 1.00 |
| Former | 577 | 4.18 | 1.14 (1.04, 1.25) |  | 95 | 0.75 | 1.27 (1.01, 1.60) |  | 462 | 3.35 | 1.07 (0.97, 1.19) |
| Current (cigarettes or equivalents/day) |  |  |  |  |  |  |  |  |  |  |  |
| <15 | 2,167 | 4.25 | 1.32 (1.25, 1.41) |  | 419 | 0.91 | 1.52 (1.31, 1.75) |  | 1,780 | 3.49 | 1.18 (1.11, 1.26) |
| 15-24 | 2,505 | 3.91 | 1.51 (1.42, 1.61) |  | 322 | 0.55 | 1.48 (1.26, 1.74) |  | 1,758 | 2.74 | 1.16 (1.08, 1.24) |
| ≥25 | 1,236 | 4.92 | 1.76 (1.63, 1.90) |  | 110 | 0.49 | 1.27 (1.01, 1.60) |  | 703 | 2.80 | 1.11 (1.01, 1.22) |
| **Alcohol intake** |  |  |  |  |  |  |  |  |  |  |  |
| Non-daily | 10,414 | 2.36 | 1.00 |  | 1,591 | 0.39 | 1.00 |  | 9,093 | 2.06 | 1.00 |
| Current daily (g of pure alcohol/day) |  |  |  |  |  |  |  |  |  |  |  |
| <15 | 109 | 4.69 | 1.01 (0.83, 1.22) |  | 18 | 0.85 | 0.84 (0.53, 1.35) |  | 82 | 3.53 | 0.87 (0.69, 1.08) |
| 15-29 | 389 | 4.62 | 1.12 (1.01, 1.24) |  | 54 | 0.70 | 1.04 (0.79, 1.36) |  | 239 | 2.84 | 0.91 (0.80, 1.04) |
| 30-59 | 656 | 4.57 | 1.13 (1.04, 1.23) |  | 75 | 0.57 | 1.02 (0.80, 1.30) |  | 402 | 2.80 | 0.97 (0.88, 1.08) |
| ≥60 | 1,052 | 5.57 | 1.48 (1.38, 1.59) |  | 95 | 0.55 | 1.18 (0.94, 1.47) |  | 582 | 3.08 | 1.14 (1.04, 1.25) |
| **Physical activity** |  |  |  |  |  |  |  |  |  |  |  |
| Quintile 1 | 2,883 | 2.99 | 1.00 |  | 471 | 0.53 | 1.00 |  | 2,804 | 2.91 | 1.00 |
| Quintile 2 | 2,523 | 2.60 | 0.92 (0.87, 0.97) |  | 423 | 0.47 | 0.87 (0.76, 1.00) |  | 2,157 | 2.22 | 0.79 (0.75, 0.84) |
| Quintile 3 | 2,453 | 2.54 | 0.89 (0.84, 0.94) |  | 322 | 0.36 | 0.65 (0.56, 0.76) |  | 1,932 | 2.00 | 0.70 (0.66, 0.74) |
| Quintile 4 | 2,455 | 2.52 | 0.87 (0.82, 0.92) |  | 348 | 0.38 | 0.64 (0.55, 0.74) |  | 1,824 | 1.87 | 0.64 (0.61, 0.69) |
| Quintile 5 | 2,306 | 2.35 | 0.80 (0.76, 0.85) |  | 269 | 0.30 | 0.50 (0.42, 0.59) |  | 1,681 | 1.71 | 0.61 (0.57, 0.65) |
| **Diet score** |  |  |  |  |  |  |  |  |  |  |  |
| 0-1 | 1,792 | 2.92 | 1.00 |  | 367 | 0.64 | 1.00 |  | 1,564 | 2.55 | 1.00 |
| 2 | 6,272 | 2.70 | 0.94 (0.89, 0.99) |  | 919 | 0.43 | 0.89 (0.78, 1.01) |  | 5,411 | 2.33 | 0.94 (0.89, 1.00) |
| 3 | 3,625 | 2.40 | 0.92 (0.86, 0.98) |  | 437 | 0.31 | 0.80 (0.68, 0.94) |  | 2,834 | 1.88 | 0.88 (0.82, 0.95) |
| 4 | 842 | 2.27 | 0.90 (0.82, 0.98) |  | 100 | 0.29 | 0.87 (0.68, 1.10) |  | 527 | 1.42 | 0.78 (0.70, 0.87) |
| 5 | 89 | 2.21 | 0.83 (0.67, 1.03) |  | 10 | 0.26 | 0.79 (0.42, 1.50) |  | 62 | 1.54 | 0.86 (0.66, 1.11) |
| **Body weight and fat** |  |  |  |  |  |  |  |  |  |  |  |
| BMI <18.5 | 981 | 4.85 | 1.32 (1.22, 1.41) |  | 356 | 2.06 | 2.15 (1.87, 2.47) |  | 992 | 4.90 | 1.25 (1.16, 1.35) |
| BMI 18.5-27.9, WC <90(M)/85(F) | 8,715 | 2.51 | 1.00 |  | 1,074 | 0.33 | 1.00 |  | 6,918 | 1.99 | 1.00 |
| BMI 18.5-27.9, WC ≥90(M)/85(F) | 1,818 | 2.68 | 0.99 (0.93, 1.05) |  | 269 | 0.42 | 1.37 (1.17, 1.60) |  | 1,558 | 2.30 | 1.29 (1.21, 1.37) |
| BMI ≥28.0, WC <90(M)/85(F) | 102 | 1.85 | 1.03 (0.85, 1.26) |  | 12 | 0.23 | 1.91 (1.07, 3.39) |  | 76 | 1.38 | 1.19 (0.95, 1.50) |
| BMI ≥28.0, WC ≥90(M)/85(F) | 1,004 | 2.27 | 0.99 (0.91, 1.07) |  | 122 | 0.29 | 1.71 (1.35, 2.15) |  | 854 | 1.93 | 1.55 (1.42, 1.70) |

Abbreviations: PY, person-year; BMI, body mass index; WC, waist circumference.

Multivariable model was adjusted for sex, education, marital status, family histories of cancer (adjusted for in cancer mortality), and hip circumference at baseline. All five lifestyle factors were included simultaneously in the same model.

**Table S4. Multivariable-adjusted hazard ratios for all-cause and cause-specific mortality by number of healthy lifestyle factors after additional adjustments or further exclusions**

|  | **No. of deaths** | **Main model^*^** | **Additional adjustment for income and occupation** | **Additional adjustment for hypertension and diabetes** | **Excluding underweight participants** | **Excluding deaths during the first two years of follow-up** |
| --- | --- | --- | --- | --- | --- | --- |
| **All-cause mortality** |  |  |  |  |  |  |
| 0 | 465 | 1.00 | 1.00 | 1.00 | 1.00 | 1.00 |
| 1 | 3,916 | 0.83 (0.75, 0.91) | 0.82 (0.75, 0.91) | 0.85 (0.77, 0.94) | 0.88 (0.78, 1.00) | 0.82 (0.74, 0.91) |
| 2 | 12,564 | 0.65 (0.59, 0.72) | 0.65 (0.60, 0.72) | 0.68 (0.62, 0.75) | 0.72 (0.64, 0.81) | 0.64 (0.58, 0.71) |
| 3 | 14,549 | 0.51 (0.46, 0.56) | 0.52 (0.47, 0.57) | 0.54 (0.49, 0.59) | 0.57 (0.51, 0.64) | 0.51 (0.46, 0.56) |
| 4 | 6,078 | 0.41 (0.38, 0.46) | 0.44 (0.40, 0.48) | 0.45 (0.40, 0.49) | 0.47 (0.42, 0.53) | 0.42 (0.38, 0.47) |
| 5 | 298 | 0.32 (0.28, 0.37) | 0.34 (0.30, 0.40) | 0.35 (0.30, 0.40) | 0.37 (0.31, 0.43) | 0.33 (0.28, 0.38) |
| **Ischaemic heart disease** |  |  |  |  |  |  |
| 0 | 43 | 1.00 | 1.00 | 1.00 | 1.00 | 1.00 |
| 1 | 565 | 1.29 (0.95, 1.76) | 1.29 (0.95, 1.77) | 1.37 (1.00, 1.87) | 1.40 (0.96, 2.03) | 1.21 (0.89, 1.66) |
| 2 | 1,822 | 0.97 (0.72, 1.32) | 0.98 (0.72, 1.33) | 1.04 (0.77, 1.41) | 1.06 (0.74, 1.53) | 0.90 (0.66, 1.22) |
| 3 | 1,981 | 0.75 (0.55, 1.02) | 0.77 (0.57, 1.05) | 0.82 (0.60, 1.11) | 0.83 (0.58, 1.20) | 0.70 (0.51, 0.96) |
| 4 | 674 | 0.58 (0.42, 0.79) | 0.62 (0.45, 0.84) | 0.65 (0.48, 0.89) | 0.65 (0.45, 0.94) | 0.55 (0.40, 0.75) |
| 5 | 31 | 0.42 (0.26, 0.67) | 0.44 (0.28, 0.71) | 0.47 (0.29, 0.74) | 0.47 (0.28, 0.78) | 0.36 (0.22, 0.58) |
| **Ischaemic stroke** |  |  |  |  |  |  |
| 0 | 19 | 1.00 | 1.00 | 1.00 | 1.00 | 1.00 |
| 1 | 166 | 0.80 (0.49, 1.28) | 0.79 (0.49, 1.28) | 0.85 (0.52, 1.36) | 0.84 (0.47, 1.48) | 0.79 (0.49, 1.30) |
| 2 | 581 | 0.62 (0.39, 0.99) | 0.63 (0.39, 0.99) | 0.67 (0.43, 1.07) | 0.64 (0.37, 1.12) | 0.61 (0.38, 0.99) |
| 3 | 597 | 0.45 (0.28, 0.71) | 0.46 (0.29, 0.73) | 0.49 (0.31, 0.78) | 0.44 (0.25, 0.78) | 0.44 (0.27, 0.70) |
| 4 | 207 | 0.34 (0.21, 0.55) | 0.37 (0.23, 0.60) | 0.39 (0.24, 0.63) | 0.33 (0.18, 0.58) | 0.33 (0.20, 0.54) |
| 5 | 8 | 0.21 (0.09, 0.49) | 0.23 (0.10, 0.53) | 0.24 (0.10, 0.54) | 0.21 (0.09, 0.51) | 0.22 (0.09, 0.51) |
| **Haemorrhagic stroke** |  |  |  |  |  |  |
| 0 | 46 | 1.00 | 1.00 | 1.00 | 1.00 | 1.00 |
| 1 | 379 | 0.69 (0.51, 0.94) | 0.68 (0.50, 0.93) | 0.75 (0.56, 1.03) | 0.68 (0.46, 0.99) | 0.68 (0.49, 0.95) |
| 2 | 1,520 | 0.63 (0.47, 0.85) | 0.62 (0.46, 0.84) | 0.70 (0.52, 0.95) | 0.62 (0.43, 0.91) | 0.61 (0.44, 0.83) |
| 3 | 1,793 | 0.48 (0.36, 0.64) | 0.49 (0.36, 0.65) | 0.54 (0.40, 0.73) | 0.45 (0.31, 0.66) | 0.47 (0.34, 0.64) |
| 4 | 740 | 0.41 (0.30, 0.55) | 0.43 (0.32, 0.59) | 0.48 (0.35, 0.65) | 0.38 (0.26, 0.56) | 0.40 (0.29, 0.56) |
| 5 | 25 | 0.37 (0.22, 0.60) | 0.41 (0.25, 0.66) | 0.43 (0.26, 0.70) | 0.35 (0.20, 0.60) | 0.36 (0.21, 0.61) |
| **Cancer** |  |  |  |  |  |  |
| 0 | 189 | 1.00 | 1.00 | 1.00 | 1.00 | 1.00 |
| 1 | 1,320 | 0.77 (0.66, 0.89) | 0.77 (0.66, 0.89) | 0.77 (0.66, 0.89) | 0.87 (0.72, 1.04) | 0.79 (0.67, 0.93) |
| 2 | 3,959 | 0.61 (0.52, 0.70) | 0.61 (0.52, 0.70) | 0.61 (0.52, 0.70) | 0.69 (0.58, 0.83) | 0.62 (0.53, 0.73) |
| 3 | 4,747 | 0.51 (0.44, 0.59) | 0.51 (0.44, 0.59) | 0.51 (0.44, 0.59) | 0.58 (0.48, 0.69) | 0.52 (0.44, 0.61) |
| 4 | 2,275 | 0.46 (0.39, 0.53) | 0.46 (0.40, 0.54) | 0.46 (0.39, 0.54) | 0.52 (0.43, 0.63) | 0.49 (0.42, 0.58) |
| 5 | 130 | 0.36 (0.29, 0.45) | 0.37 (0.29, 0.46) | 0.36 (0.29, 0.46) | 0.41 (0.32, 0.53) | 0.38 (0.30, 0.49) |
| **Respiratory diseases** |  |  |  |  |  |  |
| 0 | 29 | 1.00 | 1.00 | 1.00 | 1.00 | 1.00 |
| 1 | 225 | 0.65 (0.44, 0.96) | 0.64 (0.43, 0.95) | 0.66 (0.45, 0.98) | 0.68 (0.39, 1.20) | 0.63 (0.42, 0.96) |
| 2 | 630 | 0.42 (0.29, 0.62) | 0.43 (0.29, 0.62) | 0.43 (0.30, 0.63) | 0.52 (0.30, 0.89) | 0.42 (0.28, 0.62) |
| 3 | 705 | 0.30 (0.20, 0.44) | 0.31 (0.21, 0.45) | 0.31 (0.21, 0.45) | 0.41 (0.24, 0.70) | 0.31 (0.21, 0.46) |
| 4 | 226 | 0.19 (0.13, 0.29) | 0.21 (0.14, 0.31) | 0.20 (0.13, 0.30) | 0.28 (0.16, 0.48) | 0.20 (0.13, 0.30) |
| 5 | 18 | 0.26 (0.14, 0.48) | 0.29 (0.16, 0.52) | 0.27 (0.15, 0.49) | 0.38 (0.18, 0.77) | 0.27 (0.15, 0.50) |
| **Other causes** |  |  |  |  |  |  |
| 0 | 118 | 1.00 | 1.00 | 1.00 | 1.00 | 1.00 |
| 1 | 960 | 0.78 (0.64, 0.94) | 0.78 (0.64, 0.94) | 0.81 (0.67, 0.98) | 0.81 (0.64, 1.02) | 0.75 (0.61, 0.92) |
| 2 | 3,320 | 0.65 (0.54, 0.79) | 0.66 (0.55, 0.79) | 0.69 (0.58, 0.83) | 0.69 (0.55, 0.87) | 0.63 (0.52, 0.76) |
| 3 | 4,156 | 0.54 (0.45, 0.65) | 0.55 (0.46, 0.67) | 0.58 (0.48, 0.70) | 0.57 (0.45, 0.72) | 0.52 (0.43, 0.64) |
| 4 | 1,765 | 0.42 (0.35, 0.51) | 0.46 (0.38, 0.56) | 0.47 (0.39, 0.57) | 0.46 (0.36, 0.58) | 0.43 (0.35, 0.52) |
| 5 | 79 | 0.29 (0.22, 0.39) | 0.32 (0.24, 0.43) | 0.32 (0.24, 0.43) | 0.32 (0.23, 0.43) | 0.30 (0.23, 0.41) |

^*^Multivariable model was adjusted for sex, education, marital status, family histories of heart attack, stroke or cancer (adjusted for in all-cause mortality and corresponding cause of death), and hip circumference at baseline.

**Table S5. Subgroup analysis of population attributable risk percent for all-cause mortality by specific combination of healthy lifestyle factors**

|  | **Non-smoking, being physically active, healthy dietary habits** | **+ Healthy body weight and fat** | **+ Non-excessive alcohol intake** |
| --- | --- | --- | --- |
| **By sex** |  |  |  |
| Men | 33.2 (25.1, 40.7) | 38.0 (30.2, 45.3) | 39.4 (31.3, 47.0) |
| Women | 29.3 (21.8, 36.4) | 35.3 (27.8, 42.4) | NA |
| **By residence** |  |  |  |
| Urban | 32.5 (25.9, 38.8) | 37.5 (30.6, 44.1) | 38.4 (31.3, 45.1) |
| Rural | 32.5 (22.7, 41.7) | 38.0 (28.7, 46.7) | 38.6 (29.0, 47.5) |
| **By age (year)** |  |  |  |
| < 50 | 26.4 (10.2, 41.2) | 31.5 (15.5, 45.9) | 34.4 (18.2, 48.7) |
| 50-59 | 33.3 (21.8, 43.8) | 38.8 (27.6, 48.9) | 40.2 (28.7, 50.5) |
| ≥ 60 | 32.4 (25.5, 38.9) | 38.0 (31.3, 44.4) | 38.1 (31.1, 44.7) |
| **By education** |  |  |  |
| Illiterate and primary school | 31.5 (23.8, 38.8) | 37.0 (29.5, 43.9) | 37.3 (29.6, 44.5) |
| Middle school | 30.9 (21.8, 39.5) | 36.6 (27.4, 45.2) | 38.4 (29.0, 47.1) |
| College and university | 38.7 (19.3, 55.2) | 43.8 (23.0, 60.8) | 44.8 (23.6, 62.0) |
| **By household income (RMB/year)** |  |  |  |
| <10,000 | 27.7 (13.1, 41.1) | 33.2 (19.3, 45.8) | 33.5 (19.2, 46.4) |
| 10,000-19,999 | 32.9 (22.7, 42.3) | 38.3 (28.2, 47.5) | 39.2 (28.9, 48.7) |
| ≥20,000 | 28.5 (20.7, 36.0) | 35.0 (27.1, 42.5) | 36.3 (28.1, 44.0) |
| **By family history of chronic diseases^*^** |  |  |  |
| No | 32.0 (25.3, 38.5) | 37.6 (31.0, 43.9) | 38.3 (31.5, 44.7) |
| Yes | 32.1 (22.8, 40.9) | 37.5 (28.1, 46.1) | 38.5 (28.9, 47.4) |
| **By baseline status of hypertension and diabetes** |  |  |  |
| Neither hypertension nor diabetes | 26.6 (17.3, 35.5) | 31.9 (22.7, 40.5) | 32.7 (23.3, 41.6) |
| Either hypertension or diabetes or both | 35.5 (28.8, 41.9) | 40.6 (33.9, 46.9) | 41.1 (34.1, 47.5) |

Abbreviations: PAR%, population attributable risk percent; NA, not available.

Multivariable model was adjusted for sex, education, marital status, family histories of heart attack, stroke, and cancer, menopausal status (for women only), and hip circumference at baseline. All five lifestyle factors were included simultaneously in the same model.

^*^Participants with a family history of acute myocardial infarction, stroke, or cancer were considered as having a family history of chronic diseases.

**Table S6. Multivariable-adjusted population attributable risk percent for all-cause and cause-specific mortality by specific combination of healthy lifestyle factors among some subpopulations**

|  | **All-cause mortality** | **Ischaemic heart disease** | **Ischaemic stroke** | **Haemorrhagic stroke** | **Cancer** | **Respiratory diseases** | **Other causes** |
| --- | --- | --- | --- | --- | --- | --- | --- |
| **Never-regular (daily) smokers** |  |  |  |  |  |  |  |
| Physically active, healthy dietary habits | 27.0 (20.5, 33.2) | 37.0 (20.3, 51.6) | 31.9 (0.6, 57.5) | 35.0 (12.5, 54.0) | 11.4 (-0.3, 22.8) | 28.3 (-2.7, 54.4) | 32.0 (20.5, 42.6) |
| + Healthy body weight and fat | 33.2 (26.7, 39.4) | 44.5 (28.5, 58.2) | 36.7 (4.7, 61.9) | 40.1 (18.0, 58.3) | 13.5 (0.7, 25.9) | 41.0 (12.4, 63.3) | 37.7 (26.3, 48.1) |
| + Non-excessive alcohol intake | 33.3 (26.7, 39.5) | 44.7 (28.5, 58.4) | NA | 40.2 (17.9, 58.5) | 13.9 (0.9, 26.4) | 41.2 (12.4, 63.7) | NA |
| **Excluding underweight participants** |  |  |  |  |  |  |  |
| Non-smoking, physically active, healthy dietary habits | 31.9 (26.3, 37.3) | 36.1 (21.4, 49.2) | 43.8 (18.9, 63.5) | 35.4 (14.8, 53.0) | 22.5 (12.8, 31.8) | 32.2 (5.0, 55.0) | 31.9 (21.0, 41.9) |
| + Healthy body weight and fat | 35.3 (29.6, 40.9) | 43.1 (28.8, 55.4) | 48.6 (23.8, 67.5) | 40.1 (20.0, 57.0) | NA | 37.6 (10.2, 59.6) | 36.2 (25.3, 46.2) |
| + Non-excessive alcohol intake | 36.1 (30.2, 41.8) | NA | 49.0 (23.5, 68.2) | 41.5 (20.9, 58.5) | 24.8 (14.8, 34.3) | 37.8 (9.4, 60.5) | 36.4 (25.1, 46.8) |
| **Excluding deaths during the first two years of follow-up** |  |  |  |  |  |  |  |
| Non-smoking, physically active, healthy dietary habits | 31.6 (25.8, 37.1) | 35.1 (20.0, 48.5) | 45.9 (21.2, 65.1) | 34.2 (12.9, 52.5) | 21.4 (11.2, 31.1) | 31.2 (4.4, 53.8) | 31.4 (20.3, 41.8) |
| + Healthy body weight and fat | 36.8 (31.1, 42.3) | 42.3 (27.8, 54.9) | 49.9 (25.3, 68.5) | 37.4 (16.2, 55.4) | 22.6 (11.7, 33.0) | 43.7 (19.8, 62.7) | 36.5 (25.5, 46.6) |
| + Non-excessive alcohol intake | 37.8 (31.9, 43.4) | 42.4 (27.5, 55.3) | 50.5 (25.2, 69.3) | 39.1 (17.4, 57.1) | 25.2 (14.1, 35.7) | 44.1 (19.3, 63.6) | 36.9 (25.5, 47.3) |

Abbreviations: PAR%, population attributable risk percent; NA, not available.

Multivariable model was adjusted for sex, education, marital status, family histories of heart attack, stroke or cancer (adjusted for in all-cause mortality and corresponding cause of death), and hip circumference at baseline. All five lifestyle factors were included simultaneously in the same model.
